# Study of genetic parameters for pre-weaning growth traits in inner Mongolia white Arbas cashmere goats

**DOI:** 10.3389/fvets.2022.1026528

**Published:** 2023-01-10

**Authors:** Ruijun Wang, Yan Liu, Yue Shi, Yunpeng Qi, Yanbo Li, Zhiying Wang, Yanjun Zhang, Yanhong Zhao, Rui Su, Jinquan Li

**Affiliations:** ^1^College of Animal Science, Inner Mongolia Agricultural University, Hohhot, Inner Mongolia, China; ^2^College of Vocational and Technical, Inner Mongolia Agricultural University, Baotou, Inner Mongolia, China

**Keywords:** genetic parameters, fixed effects, variance components, pre-weaning growth traits, inner Mongolia cashmere goats

## Abstract

Inner Mongolia Arbas white cashmere goats is a dual-purpose breed for producing cashmere and meat. In recent years, its meat has become more and more popular among consumers because of rich nutrients and delicious flavor. Therefore, it is particularly important to study the genetic and non-genetic factors affecting the early growth traits and estimate variance components of pre-weaning growth traits of Inner Mongolia Albas white cashmere goats. A total of 37487 kidding records such as birth weight (BWT), weaning weight (WWT), average daily gain from birth to weaning (ADG) and Kleiber ratio (KR) from 343 sires and 7296 dams were used in this study. The most appropriate model was chosen on the basis of likelihood ratio test by fitting six models which excluding or including maternal genetic, maternal permanent environmental effects. The parameters were estimated under the most appropriate model using AIREML method by WOMBAT software. With the best model (Model 6), heritability estimates were 0.0435, 0.0911, 0.0932 and 0.2339 for BWT, WWT, ADG and KR traits, respectively. Maternal heritability estimates were 0.0143, 0.0246, 0.0220 and 0.0186 for BWT, WWT, ADG, and KR traits respectively. The correlation between different traits was estimated with the most suitable model by using bivariate analysis method. The direct additive genetic correlation among the traits ranged from −0.026 (BWT~KR) to 0.772 (ADG~KR). The maternal permanent environment correlation is between −0.289 (BWT-KR) ~0.900 (WWT-ADG). Results indicated that maternal effects and direct-maternal genetic covariance should be considered in any program aimed at improving pre-weaning growth traits to have an accurate genetic evaluation. In addition, positive and medium to high genetic correlations generally exist among WWT, ADG and KR due to the existence of genetic variation for early growth traits. The results showed that the genetic progress of these traits could be slowly through selection except for KR.

## 1. Introduction

Goat numbers in China have been estimated at ~133.5 million, accounting for 10.6% of the world's goat population (FAOSTAT, https://www.fao.org/faostat/en/). As is well-known, goats are important multipurpose economic animals. Cashmere goat is a breed mainly raised for cashmere production. Cashmere goats live primarily in the arid and semi-arid areas of China (1). There is a unique natural prairie in the Inner Mongolia Autonomous Region that has become a very important livestock production base in China (2). The major breeds of cashmere goats are the Inner Mongolia white cashmere goats, the Hanshan white cashmere goats, and the Ujimqin white goats. The Inner Mongolia white cashmere goat breed includes three groups: Arbas, Alxa, and Erlangshan.

The Inner Mongolia Arbas white cashmere goat (IMWACGs) is a dual-purpose breed for producing meat and cashmere, which is mainly distributed in Ordos, Inner Mongolia, China. Traditionally, IMWACGs mainly produced high-quality cashmere, supplemented by meat production. In the last decade, cashmere profits have fallen whereas mutton profits have gradually increased. Goat meat is valued as a high-protein and low-fat food that contains fatty acids and amino acids necessary for the human body (3). Therefore, goat meat has won the favor and praise of many customers. Currently, the price per kilogram of a carcass of an IMWACG has reached ¥90, which has doubled in the last 10 years.

An understanding of the influencing factors and the genetic principles affecting the growth traits is necessary to implement optimal breeding and selection programs (4). Previous studies primarily focused on certain traits of IMWACGs, such as cashmere quality, cashmere yield, and yearling and adult weights, the weight after shearing (2, 5–9), and rarely or even neglected early growth traits (10), which have a strong correlation with the mature weight (11). Furthermore, rapid growth during the pre-weaning period may reduce the rearing costs to a minimum and bring more interest to the farmers (12).

Early growth is influenced by several factors, including environmental variables such as herd, grassland quality, direct genetic effects of the kid, and the maternal additive genetic and permanent environmental effects of the dam (13). Several studies on various breeds have documented the importance of these sources of variation on the early growth traits of goats (4, 12, 14–18).

This study aimed at estimating the genetic parameters for preweaning body weight traits and Kleiber ratio and also the genetic correlations among these traits to consider these traits in the genetic selection program.

## 2. Materials and methods

### 2.1. Animals and data collection

The IMWACG population is mostly raised in Ordos (Inner Mongolia, China), the environmental details of which have been described by Zhou et al. (10) and Bai et al. (5). Data and pedigree information were collected from 1998 to 2018 by the Inner Mongolia Yiwei White Cashmere Goat Co., Ltd. Outliers ($|x|>\bar{x}\pm 3s.d$, *x* indicates the observational value of traits) and records from animals from an unknown dam were omitted. Finally, 37,487 kidding records from 343 sires and 7,296 dams were used in this study. The kids were weighed and ear-tagged at birth and weaned for about 90 days. The kids were fed with hay freely during the day and suckled with the grazed dams during the night until 30 days of age. 2 months after birth, the kids suckled their mothers two times a day, morning and evening, until weaning. The maiden does were mated by artificial insemination at about 18 months of age. In the present study, the birth weight (BWT), average daily gain from birth to weaning (ADG), weaning weight (WWT), and preweaning Kleiber ratio (KR) were analyzed. The Kleiber ratio is an indirect selection method for measuring feed conversion. The data used in the analysis are given in Table 1. The Kleiber ratio (KR) for preweaning was calculated as follows (19):


$$\begin{array}{l} KR=\frac{ADG}{WWT^{0.75}} \end{array}$$

**Table 1:** Characteristics of the data structure for early growth traits of IMWACGs.

**Item**	**BWT, kg**	**WWT, kg**	**ADG, g**	**KR**
No. of records	37,487	26,181	26,181	26,181
No. of animals	40,012	28,879	28,879	28,879
No. of sires	343	322	322	322
No. of dams	7,296	6,469	6,469	6,469
No. records per sires	109.61	81.31	81.31	81.31
No. records per dam	5.14	4.05	4.05	4.05
Mean	2.67	20.24	147.52	15.40
S.D.	0.41	3.97	33.36	2.00
C.V(%)	15.27	19.61	22.61	12.99

BWT, birth weight; WWT, weaning weight; ADG, average daily gain from birth to weaning; KR, Kleiber ratio (ADG/WWT^0.75^); C.V., coefficient of variation.

### 2.2. Statistical analysis

The fixed effects of each trait were determined by a general linear model (GLM) procedure using SAS software (20). The fixed effects include the year of kidding (1998–2018), herd (six doe flocks), birth type (single or twins), sex (male or female), and age of the dam at kidding (2–7 years old). Duncan's test was performed to compare the differences between various levels of the same significant factor. A statistical model of GLM is described below. The age of kids upon weaning (in days) was used as a covariate effect for the trait of WWT.


$$\begin{array}{c} y_{ijklmn}=\mu +P_{i}+H_{j}+T_{k}+S_{l}+D_{m}+{\left(PH\right)}_{ij}+{\left(TS\right)}_{kl}\\ +\;e_{ijklmn}, \end{array}$$

where *y*_*ijklmn*_ is the observation of *nth* kids that belonged to the *ith* year, the *jth* herd, the *kth* type of birth, the *lth* sex, and the *mth* dam age. μ is the overall mean, (*PH*)_*ij*_ is the interaction between the *ith* year and the *jth* herd, (*TS*)_*kl*_ is the interaction between the *kth* type of birth and the *lth* sex, and *e*_*ijklmn*_ is the random error.

The (co)variance components were estimated for each trait by the restricted maximum likelihood (REML) method using the WOMBAT program (21). Six different models were created for each trait, by excluding or including the maternal additive genetic effect, maternal permanent environmental effect, and the covariance between the direct-maternal additive genetic effect. Convergence is achieved when the change in log likelihood between the last two iterations is <10^−8^. The models are as follows:


(1) $$\begin{array}{c} y=Xb+Z_{1}a+e \end{array}$$

(2) $$\begin{array}{c} y=Xb+Z_{1}a+Z_{3}c+e \end{array}$$

(3) $$\begin{array}{c} y=Xb+Z_{1}a+Z_{2}m+eCov\left(a,m\right)=0 \end{array}$$

(4) $$\begin{array}{c} y=Xb+Z_{1}a+Z_{2}m+eCov\left(a,m\right)=A\sigma_{am} \end{array}$$

(5) $$\begin{array}{c} y=Xb+Z_{1}a+Z_{2}m+Z_{3}c+e\;Cov\left(a,m\right)=0 \end{array}$$

(6) $$\begin{array}{c} y=Xb+Z_{1}a+Z_{2}m+Z_{3}c+e\;Cov\left(a,m\right)=A\sigma_{am}, \end{array}$$

where y is a vector of observations on the different traits; and b, a, m, c, and e are the vectors of the fixed effects, the direct additive genetic effects, the maternal additive genetic effects, the maternal permanent environmental effects, and the residual effects, respectively. X, Z_1_, Z_2_, and Z_3_ are the design matrices associating the fixed effects, the direct additive genetic effects, the maternal additive genetic effects, and the maternal permanent environmental effects to the *y* vector. For WWT, we also considered the days between birth to weaning as the covariate for quadratic regression in the model.

The direct additive genetic effects, the maternal additive genetic effects, the maternal permanent environmental effects, and the residual effects are assumed to be normally distributed with mean 0 and $Var\left(a\right)=A\sigma_{a}^{2}$, $Var\left(m\right)=A\sigma_{m}^{2}$, $Var\left(p\right)=I_{d}\sigma_{c}^{2}$, and $Var\left(e\right)=I_{n}\sigma_{e}^{2}$, respectively, whereas $\sigma_{a}^{2}$, $\sigma_{m}^{2}$, $\sigma_{c}^{2}$, and $\sigma_{e}^{2}$ are the direct additive genetic variance, the maternal additive genetic variance, the maternal permanent environmental variance, and the residual variance, respectively. *A* is the additive genetic correlation matrix, *I*_*d*_ and *I*_*n*_ are the identity matrices that have an order equal to the number of dams and kids, respectively, and σ_*am*_ denotes the covariance between the direct additive genetic and the maternal additive genetic effects.

To create the most suitable model for the traits, a likelihood ratio test (LR) was performed to obtain the appropriate model as given below (22):


$$\begin{array}{c} LR=-2\log\frac{L_{1}}{L_{2}}=-2\left[\log\left(L_{1}\right)\right]-\left[\log\left(L_{2}\right)\right], \end{array}$$

where L_1_ and L_2_ are the maximum likelihood function values of model 1 and model 2, respectively. Model 1 is a sub-model of model 2. LR obeys the chi-square distribution. The degree of freedom is the number of parameters considered in model 2 minus the number of parameters in model 1.

Furthermore, the genetic and phenotypic correlations between the traits were obtained using bivariate animal models based on the most appropriate model for each trait.

## 3. Results

### 3.1. Fixed effects

From Table 1, the coefficient of variation of BWT, WWT, ADG, and KR reached 15.27, 19.61, 22.61, and 12.99%, respectively. Table 2 shows the least square mean of each trait and its standard deviation. The results showed that sex, birth type, birth herds, birth year, birth month, and age of dam have a very significant impact on each trait. The interaction of sex and birth type had a significant impact on weaning weight and daily gain. Therefore, sex and birth type are combined to form a new variable s_t in the model for estimating these two traits, which is composed of different levels of gender and birth type. For all traits, the interaction among year, group, and month of birth was significant. Therefore, they could be combined into a new variable h_ y_ m in the model. This variable was composed of different years, groups, and months at birth. Finally, the fixed effects of each character were determined as shown in Table 3.

**Table 2:** Least-squares *means* ± S.D. for the studied traits.

**Component**	**BWT, kg**		**WWT, kg**		**ADG, g**		**KR**	
	***n***	$\bar{x}$	***n***	$\bar{x}$	***n***	$\bar{x}$	***n***	$\bar{x}\pm S$
**Model created**		^**^		^**^		^**^		^**^
**Sex**		^**^		^**^		^**^		^**^
Male	18,684	2.70 ± 0.42	9,862	22.47 ± 4.0	9,862	166.59 ± 32.66	9,862	16.10 ± 1.76
Female	18,803	2.65 ± 0.40	16,319	18.90 ± 3.29	16,319	136.05 ± 28.10	16,319	14.97 ± 1.94
**Birth type**		^**^		^**^		^**^		^**^
Single	14,238	2.76 ± 0.43	11,574	21.66 ± 3.97	11,574	162.4 ± 32.51	11,574	16.18 ± 2.09
Twin	23,249	2.62 ± 0.38	14,607	19.11 ± 3.59	14,607	135.71 ± 29.01	14,607	14.78 ± 1.58
**Herd**		^**^		^**^		^**^		^**^
1	6,215	2.66 ± 0.41^bc^	4,392	19.58 ± 4.14^e^	4,392	143.12 ± 34.17^e^	4,392	15.31 ± 2.02^c^
2	6,292	2.68 ± 0.41^bc^	4,260	19.99 ± 3.81^d^	4,260	144.68 ± 32.14^d^	4,260	15.24 ± 1.87^d^
3	6,423	2.68 ± 0.42^b^	4,554	20.67 ± 3.96^b^	4,554	150.95 ± 33.02^b^	4,554	15.52 ± 1.94^a^
4	6,193	2.70 ± 0.41^a^	4,469	20.83 ± 3.97^a^	4,469	152.45 ± 33.67^a^	4,469	15.56 ± 1.88^a^
5	6,379	2.67 ± 0.41^bc^	4,445	20.34 ± 3.83^c^	4,445	148.70 ± 32.95^c^	4,445	15.46 ± 1.95^b^
6	5,985	2.66 ± 0.39^c^	4,061	19.99 ± 3.97^d^	4,061	144.89 ± 33.06^d^	4,061	15.27 ± 2.05^cd^
**Year**		^**^		^**^		^**^		^**^
Max	1,759	2.78 ± 0.40(2007)	1,151	22.97 ± 4.00(2,012)	1,151	171.94 ± 33.39(2012)	1,151	17.07 ± 2.44(2010)
Min	2,024	2.56 ± 0.37(2011)	1,324	15.41 ± 2.60(2,000)	1,324	108.74 ± 23.16(2,000)	1,324	13.92 ± 1.84(2000)
**Month**		^**^		^**^		^**^		^**^
1	87	2.67 ± 0.40^a^	55	21.40 ± 3.78^a^	55	99.10 ± 21.40^d^	55	9.88 ± 1.00^e^
2	168	2.69 ± 0.41^a^	113	20.89 ± 4.33^ab^	113	119.17 ± 30.11^c^	113	12.05 ± 1.37^d^
3	29,179	2.68 ± 0.42^a^	20,920	20.76 ± 3.89^b^	20,920	146.80 ± 32.41^b^	20,920	14.98 ± 1.41^c^
4	7,174	2.67 ± 0.38^a^	4538	18.51 ± 3.38^c^	4,538	151.34 ± 35.41^a^	4,538	16.83 ± 2.17^b^
5	879	2.66 ± 0.36^a^	555	14.68 ± 3.22^d^	555	155.15 ± 42.78^a^	555	20.55 ± 3.35^a^
**Dam age**		^**^		^**^		^**^		^**^
2	6,741	2.65 ± 0.41^c^	4,413	19.73 ± 3.86^d^	4,413	147.17 ± 32.54^bc^	4,413	15.68 ± 2.04^a^
3	7,408	2.67 ± 0.41^ab^	5,181	20.16 ± 4.07^c^	5,181	147.12 ± 33.99^bc^	5,181	15.40 ± 1.96^b^
4	7,244	2.69 ± 0.41^ab^	5,170	20.37 ± 4.04^b^	5,170	147.90 ± 34.15^ab^	5,170	15.36 ± 1.98^bc^
5	6,633	2.68 ± 0.40^ab^	4,796	20.53 ± 3.86^a^	4,796	148.37 ± 32.67^a^	4,796	15.31 ± 1.89^cd^
6	5,705	2.69 ± 0.41^a^	4,104	20.25 ± 3.98^c^	4,104	146.60 ± 33.58^c^	4,104	15.30 ± 1.98^d^
7 or more	3,756	2.67 ± 0.41^b^	2,517	20.49 ± 3.94^a^	2,517	148.34 ± 32.75^a^	2,517	15.33 ± 1.75^cd^
Sex^*^type		ns		^**^		^**^		ns
Herd^*^year^*^month		^**^		^**^		^**^		^**^

BWT, birth weight; ADG, average daily gain from birth to weaning; WWT, weaning weight; KR, Kleiber ratio (ADG/WWT ^0.75^) from birth to weaning; ns: non-significant (*p* > 0.05). The means with different letters in each sub-class within a column differ significantly from another. ^*^p < 0.05 and

** *p* < 0.01.

**Table 3:** The fixed effects used in the models for traits.

**Traits**	**Sex**	**Birth type**	**s_t**	**h_y_m**	**Dam age**
BWT	✓	✓		✓	✓
WWT			✓	✓	✓
ADG			✓	✓	✓
KR	✓	✓		✓	✓

BWT, birth weight; ADG, average daily gain from birth to weaning; WWT, weaning weight; KR, Kleiber ratio (ADG/WWT ^0.75^) from birth to weaning; s_t, A combination of different levels of sex and birth type; h_y_m:A combination of the group, year, and month at birth.

### 3.2. Model comparisons

The variance groups of BWT, WWT, ADG, and KR estimated by the six models are listed in Table 4. The variance components estimated by different models for the same trait were quite different. All traits showed that the additive genetic variance and heritability (h^2^) estimated by model 1 were the highest. With the inclusion of the maternal genetic effect, the maternal environmental effect, or the individual additive and maternal additive genetic covariance in other models, the direct additive genetic variance and heritability estimates decreased.

**Table 4:** Estimates of the (co)variance components and the genetic parameters studied for the traits.

**Traits**	**Models**	** ${\sigma_{\alpha }}^{2}$ **	** ${\sigma_{m}}^{2}$ **	**σ_*a*_*m***	** ${\sigma_{c}}^{2}$ **	** ${\sigma_{e}}^{2}$ **	** ${\sigma_{p}}^{2}$ **	** ${h_{d}}^{2}$ **	** ${h_{m}}^{2}$ **	***c* ^2^**	***r*_*am*_**	**−2*LogL***
BWT	Model 1	0.0151				0.1432	0.1583	0.0954 ± 0.01				−30855.36
	Model 2	0.0047			0.0093	0.1418	0.1558	0.0302 ± 0.01		0.0598 ± 0.00		−31084.50
	Model 3	0.0045	0.008			0.1446	0.1572	0.0292 ± 0.01	0.0511 ± 0.01			−30979.46
	Model 4	0.0069	0.0118	– 0.0043		0.1432	0.1576	0.0443 ± 0.01	0.0751 ± 0.01		−0.4802 ± 0.11	−30988.84
	Model 5	0.0044	0.001		0.0085	0.1419	0.1559	0.0284 ± 0.01	0.0064 ± 0.00	0.0551 ± 0.01		−31087.86
	**Model 6**	**0.0068**	**0.0022**	**– 0.0024**	**0.0089**	**0.1406**	**0.1561**	**0.0435** **±0.01**	**0.0143** **±0.01**	**0.0567** **±0.01**	**−0.6097** **±0.14**	**−31095.68**
WWT	Model 1	1.7312				4.5941	6.3253	0.2737 ± 0.02				72386.53
	Model 2	1.0969			0.3125	4.7307	6.1402	0.1786 ± 0.02		0.0509 ± 0.01		72362.84
	Model 3	0.6276	0.4582			5.004	6.0898	0.1030 ± 0.02	0.0752 ± 0.01			72364.79
	Model 4	0.5119	0.2726	0.2389		5.0637	6.0871	0.0841 ± 0.01	0.0448 ± 0.01		0.6396 ± 0.14	72348.96
	Model5	0.7269	0.2374		0.1949	4.912	6.0711	0.1197 ± 0.02	0.0391 ± 0.01	0.0321 ± 0.01		72346.90
	**Model 6**	**0.5517**	**0.1492**	**0.1925**	**0.1646**	**5.0013**	**6.0593**	**0.0911** **±0.02**	**0.0246** **±0.01**	**0.0272** **±0.01**	**0.6710** **±0.16**	**72335.19**
ADG	Model 1	127.75				332.51	460.26	0.2776 ± 0.02				183122.89
	Model 2	86.249			20.93	340.98	448.16	0.1925 ± 0.02		0.0467 ± 0.01		183060.47
	Model 3	49.941	31.606			361.85	443.4	0.1126 ± 0.02	0.0713 ± 0.01			183059.37
	Model 4	37.87	17.395	19.242		368.05	442.56	0.0856 ± 0.01	0.0393 ± 0.01		0.7497 ± 0.13	183047.75
	Model 5	60.432	15.214		13.687	353.66	442.99	0.1364 ± 0.02	0.0343 ± 0.01	0.0309 ± 0.01		183039.35
	**Model 6**	**41.078**	**9.6975**	**15.779**	**10.843**	**363.5**	**440.9**	**0.0932** **±0.02**	**0.0220** **±0.01**	**0.0246** **±0.01**	**0.7906** **±0.15**	**183029.45**
KR	Model 1	0.35106				0.79058	1.1416	0.3075 ± 0.02				27806.74
	Model 2	0.275			0.0444	0.80071	1.1201	0.2455 ± 0.02		0.0396 ± 0.01		27758.10
	Model 3	0.23245	0.056			0.82917	1.1177	0.2080 ± 0.02	0.0501 ± 0.01			27765.66
	Model 4	0.2341	0.0567	– 0.0012		0.8283	1.118	0.2094 ± 0.03	0.0508 ± 0.01		−0.0103 ± 0.02	27765.66
	Model 5	0.2491	0.0187		0.0344	0.8129	1.1151	0.2234 ± 0.02	0.0168 ± 0.01	0.0308 ± 0.01		27750.58
	**Model 6**	**0.2614**	**0.0207**	**−0.0064**	**0.035**	**0.8066**	**1.1174**	**0.2339** **±0.03**	**0.0186** **±0.01**	**0.0314** **±0.01**	**−0.0874** **±0.02**	**27750.5000**

BWT: birth weight; ADG: average daily gains from birth to weaning; WWT: weaning weight; KR: Kleiber ratio (ADG/WWT0.75) from birth to weaning; $\sigma_{a}^{2}$: direct additive genetic variance; $\sigma_{m}^{2}$: maternal additive genetic variance; $\sigma_{c}^{2}$: maternal permanent environmental variance; σ_*a*_*m*: direct-maternal genetic covariance; $\sigma_{e}^{2}$: residual variance; $\sigma_{p}^{2}$: phenotypic variance; $h_{d}^{2}$: direct heritability; $h_{m}^{2}$: maternal heritability; c2: ratio of maternal permanent environmental effect; ram: direct-maternal genetic correlation; -2log L: -2*log likelihood; S.E.: standard error.

The likelihood ratio test results of the different models are listed in Table 5. Except for the BWT models 2 and 5, the KR models 3 and 4, and the KR models 5 and 6, there were significant differences among the models regarding other traits. It showed that the most suitable models of BWT, WWT, and ADG should include direct additive genetic effects, maternal genetic and environmental effects, and direct maternal additive genetic correlation. The comparison results of the different models of KR traits showed that increasing the direct and maternal genetic covariance in the model did not significantly improve the goodness-of-fit of the model and that the direct and maternal heritability had not changed much. However, considering the direct and maternal genetic covariance was negatively correlated (−0.0874), the estimated value of the direct additive genetic effect could be appropriately increased. In conclusion, model 6 has more advantages than other models in estimating the variance components and genetic parameters for all traits.

**Table 5:** Likelihood ratios and χ2 test results of different model comparisons.

**Model comparison**	**df**	**BWT**	**WWT**	**ADG**	**KR**
1:2	1	229.14^***^	23.68^***^	62.42^***^	48.64^***^
1:3	1	124.1^***^	21.74^***^	63.52^***^	41.08^***^
1:4	2	133.48^***^	37.57^***^	75.14^***^	41.08^***^
1:5	2	232.5^***^	39.62^***^	83.54^***^	56.16^***^
1:6	3	240.32^***^	51.34^***^	93.44^***^	56.24^***^
2:5	1	3.36 ns	15.94^***^	11.82^***^	7.52^***^
2:6	2	11.18^**^	27.66^***^	21.12^***^	7.60^*^
3:4	1	9.38^**^	15.83^***^	11.62^***^	0.00 ns
3:5	1	108.4^***^	17.88^***^	20.02^***^	15.08^***^
3:6	2	116.22^***^	29.60^**^	29.92^***^	15.16^***^
4:6	1	106.84^***^	13.77^***^	18.30^***^	15.16^***^
5:6	1	7.82^**^	11.72^***^	9.90^**^	0.08 ns

BWT, birth weight; ADG, average daily gain from birth to weaning; WWT, weaning weight; KR, Kleiber ratio (ADG/WWT^0.75^) from birth to weaning;

*** *P* < 0.001;

** *P* < 0.01;

* *P* < 0.05;

ns :*P* > 0.05.

### 3.3. Genetic parameter estimates

The variance components and genetic parameters of different models for each trait were estimated and are shown in Table 4. The heritability of BWT estimated by different models ranged from 0.0284 (model 5) to 0.0954 (model 1), the maternal heritability ranged from 0.0064 (model 5) to 0.0751 (model 4), and the variance ratio of the maternal permanent environmental effects ranged from 0.0551 (model 5) to 0.0598 (model 2). The direct genetic correlation with maternal heritability was −0.4802 for model 4 and −0.6097 for model 6, respectively. They were highly negatively correlated.

It was estimated that the correlation of WWT heritability is between 0.0841 (model 4) and ~0.2737 (model 1), maternal heritability is between 0.0246 (model 6) and ~0.0752 (model 3), the variance ratio of the maternal permanent environmental effects is between 0.0272 (model 6) and ~0.0509 (model 2), and that of direct genetic effect with maternal heritability is 0.6396 (model 4) and 0.6710 (model 6). They have a high positive correlation.

The ADG heritability estimated by different models was between 0.0856 (model 4) and 0.2776 (model 1), the maternal heritability range was between 0.0220 (model 6) and 0.0713 (model 3), and the variance ratio of the maternal permanent environmental effects was between 0.0246 (model 6) and 0.0467 (model 2). There was a highly positive correlation between the direct additive effect and maternal genetic correlation, which was estimated to be 0.7497 by model 4 and 0.7906 by model 6.

The KR heritability estimated by different models was between 0.2080 (model 3) and ~0.3075 (model 1). The variance ratio of maternal heritability to maternal permanent environmental effect was low, ranging from 0.0168 (model 5), ~0.0501 (model 4), 0.0308 (model 5), and ~0.0396 (model 2), respectively. The direct additive effect had a low negative correlation with the maternal genetic effect, ranging from −0.0103 (model 4) to approximately −0.0874 (model 6).

As presented in Table 4, the heritabilities of BWT, WWT, ADG, and KR were estimated to be 0.0435, 0.0911, 0.0932, and 0.2339, respectively, using optimal models for each trait (model 6), which belonged to low-to-medium heritability. Maternal heritability was 0.0143, 0.0246, 0.0220, and 0.0186, respectively, which belonged to low heritability. In addition to maternal heritability, the proportions of maternal permanent environmental effects also belonged to low heritability, which were 0.0567, 0.0272, 0.0246, and 0.0314, respectively. The correlation between the direct genetic effect and the maternal genetic effect was −0.6097, 0.6710, 0.7906, and −0.0874 for BWT, WWT, ADG, and KR, respectively. However, KR had a weak negative correlation, WWT and ADG had a strong positive correlation, and BWT had a strong negative correlation.

### 3.4. Correlation estimates

The correlation between different effects was estimated with the most suitable model of traits using the bivariate analysis method. The results are shown in Table 6. The direct additive genetic correlation among the traits ranged from −0.026 (BWT~KR) to 0.772 (ADG~KR). The absolute value of the genetic correlation between BWT and other traits was lower than 0.05. It was between −0.026 (BWT-KR) and ~0.012 (BWT-WWT), which belonged to low genetic correlation. The genetic correlation between WWT and ADG was 0.614. The maternal genetic correlation between WWT and KR was negative (−0.182) and low, and was 0.388, 0.406, and 0.270 for BWT-WWT, BWT-ADG, and BWT-KR, respectively, but was positive among other traits. The correlation was medium and high for WWT-ADG (0.577) and ADG-KR (0.703), respectively. The maternal permanent environment correlation was −0.289 for BWT-KR and ~0.900 for WWT-ADG, whereas for BWT-WWT, BWT-ADG, WWT-KR, and ADG-KR it was 0.252, 0.045, 0.308, and 0.682, respectively.

**Table 6:** Estimates of the (co)variance components and the genetic parameters for each trait using the best models by bivariate analysis.

**Trait 1**	**Trait 2**	***r*_*p*_**	***r*_*d*_**	***r*_*c*_**	***r*_*m*_**	***r*_*e*_**
BWT	WWT	0.062 ± 0.01	0.012 ± 0.15	0.252 ± 0.11	0.388 ± 0.28	0.055 ± 0.01
BWT	ADG	−0.098 ± 0.01	−0.002 ± 0.14	0.045 ± 0.12	0.406 ± 0.25	−0.123 ± 0.01
BWT	KR	−0.294 ± 0.01	−0.026 ± 0.14	−0.289 ± 0.09	0.270 ± 0.30	−0.366 ± 0.01
WWT	ADG	0.927 ± 0.00	0.614 ± 0.06	0.900 ± 0.03	0.577 ± 0.11	0.957 ± 0.00
WWT	KR	0.589 ± 0.01	−0.001 ± 0.10	0.308 ± 0.17	−0.182 ± 0.14	0.739 ± 0.02
ADG	KR	0.836 ± 0.00	0.772 ± 0.04	0.682 ± 0.10	0.703 ± 0.07	0.888 ± 0.01

BWT, birth weight; ADG, pre-weaning daily gain from birth to weaning; WWT, weaning weight; KR, pre-weaning Kleiber ratio (ADG/WWT^0.75^).

The phenotypic correlations between BWT and other traits were both low negative and positive correlations (BWT-KR: −0.294 and BWT-ADG: −0.098; BWT-WWT: 0.062) and the phenotypic correlations between other traits were highly positive correlations (WWT-KR: 0.589, WWT-ADG: 0.927, and ADG-KR: 0.836).

## 4. Discussion

### 4.1. Fixed effects

The coefficient of variation of each trait was between 12 and 23%, indicating that each character had a certain degree of phenotypic variation. It was necessary to further analyze the source of variation. The results revealed that sex, birth type, birth year and month of kids, birth herd, and age of dam have significant effects on BWT, WWT, ADG, and KR (*p* < 0.01) in the present study and were in agreement with the previous reports on Sirohi goats (23) and Raini Cashmere goats (19). The significant effects of birth year, birth month, and birth herd could be attributed to the differences in management, food availability, diseases, and the condition of the climate and raising systems, as described by Ehsaninia (24) for Sangsari sheep and Rashidi et al. (14, 15) for Kermani sheep. The significant effects of sex and birth type may be attributed to several reasons such as the difference in the endocrine systems of female and male kids. Twins have limited uterine space during pregnancy, inadequate nutrients during pregnancy and lactation, and competition for milk consumption. The significant effects of the impact of the dam age on pre-weaning growth traits are due to the differences in the maternal behavior and the mothering ability of the dam at different ages. The BWT of IMWACGs increased with age until 6 years of age. In contrast to BWT, KR decreased with age. However, WWT and ADG reached the highest at the age of 4 years and then gradually decreased. In this study, the difference was that 7-year-old kids were higher than 6-year-old kids, which may be due to the elimination of some 6-year-old kids with low production performance (4,104 6-year old kids but only 2,517 7-year-old kids).

### 4.2. Model comparisons

For all the study traits, model 1 only considered that the direct additive genetic effect had the highest estimate of heritability, which then gradually decreased with the other random effects considered in the models, such as the maternal additive genetic effect, the maternal permanent environmental effect, and the direct–maternal interaction effect. Based on LRT, model 6 was the most suitable model for all the traits. In addition, the direct additive genetic effect, the maternal additive genetic effect, the maternal permanent environmental effect, and the direct–maternal interaction effect also had an impact on the variation between BWT, WWT, ADG, and KR, respectively. Dige et al. (25) concluded that model 4 was the best model for ADG and KR, and model 6 was the best model for BWT and WWT, which were partly in agreement with the present study. In contrast, Gowane et al. (23) applied model 1 for BWT for Sirohi goats, and Barazandeh et al. (19) applied model 2 for BWT, WWT, and ADG, and model 1 for KR for Raini Cashmere goats. Different models have been used by researchers for the same traits in diverse breeds, which may be because of the differences in the genetic basis and population size of the breeds.

### 4.3. Genetic parameters

The direct heritability of BWT estimated by the optimal model was 0.0435, which belonged to low heritability. Similar results were reported by Yalcin et al. (26) and Gerstmayr et al. (27) in Angora goats, Magotra et al. (28) in Beetal goats, Menezes et al. (29) in Boer goats, and Bangar et al. (30) on Jakhrana goats. In addition, Rout et al. (31), Sarma et al. (32), Mokhtari et al. (17), and Kuthu et al. (33) estimated the direct heritability values of the BWT of Jamunapari goats, Assam Hill goats, Boer goats, and Teddy goats, which were 0.14, 0.19, 0.17, and 0.28, respectively, and belonged to medium heritability. Zhou et al. (34) and Portolano et al. (35) estimated that the direct heritability of the BWT of the Hainan black goats and Sicilian Girgentana goats were 0.45 and 0.49, respectively, and belonged to high heritability.

The direct heritability of WWT estimated by the optimal model was 0.0911, which belonged to low heritability. The reports of Gowane et al. (23) on Sirohi goats, Baneh et al. (11) on Naeini goats, and Barazandeh et al. (19) on Raini cashmere goats were consistent with the findings of this study. The direct heritability reports of WWT by Rout et al. (31) on Jamunapari goats, Menezes et al. (29) and Zhang et al. (4) on Boer goats, and Kuthu et al. (33) on Teddy goats ranged from 0.16 to 0.28, which belonged to medium heritability. However, high heritability values were estimated by Zhou et al. (34), Portolano et al. (35), and Tesema et al. (36) for Hainan black goats, for Sicilian Girgentana goats, and in Boer x Central Highland goats, which were 0.34, 0.36, and 0.50, respectively.

The direct heritability of ADG was estimated to be 0.0932 under the optimal model. The reports of Gowane et al. (23) on Sirohi goats and Zhang et al. (4) on Boer goats were consistent with this study. However, higher estimated values between 0.20–0.42 were obtained by Dig et al. (25) for Jamunapari goats, Jay et al. (37) for Mehsana goats, Al- Shorepy et al. (12) for Emirati goats, Baneh et al. (11) for Naeini goats, and Menezes et al. (29) for Boer goats.

The direct heritability of KR was 0.2339 as estimated by the optimal model and was higher than those of BWT, WWT, and KR. It was significantly higher than those reported by Bangar et al. (30) for Jakhrana goats, Tesema et al. (36) for Boer x Central Highland goats, and Barazandeh et al. (19) for Raini cashmere goats but consistent with the estimated value of Dige et al. (25) for Jamunapari goats.

Maternal effects in animals have been studied extensively in the past years both because of their economic importance in domestic mammals and their theoretical interest. The maternal heritability values of BWT, WWT, ADG, and KR estimated in this study were 0.0143, 0.0246, 0.0220, and 0.0186, respectively, which were lower than those reported by Menezes et al. (29) for Boer goats (0.05 for BWT, 0.12 for WWT, and 0.13 for ADG), Mohammadi et al. (38) for Raeini cashmere goats (0.17 for BWT and 0.07 for WWT), Zhang et al. (4) for Boer goats (0.26 for BWT and 0.16 for WWT), Buxadera et al. (39) for Creole goats (0.24 for BWT), and Dige et al. (25) for Jamunapari goats (0.11 for BWT, 0.26 for WWT, and 0.23 for ADG and KR). The lower maternal heritability and maternal permanent environmental effects (0.0567, 0.0272, 0.0246, and 0.0314 for BWT, WWT, ADG, and KR) may be because the effects considered for each trait in this study were more than those in other studies.

The correlation between the direct and maternal genetic effects between BWT and KR in this study is either high or low negative (−0.6097 and −0.0874), which was consistent with that reported by Menezes et al. (29) for Boer goats, Zhang et al. (4) for Boer goats, Buxadera et al. (39) for Creole goats, and Dige et al. (25) for Jamunapari goats. However, it was only contrary to the estimation of Sirohi goats by Gowane et al. (23). The negative correlation is an indication of how difficult it is to simultaneously improve both effects in a selection program. Tosh and Kemp (40) suggested that the antagonism between the effects of an individual's genes for growth and those of its dam for a maternal contribution might be due to natural selection for an intermediate optimum, but this is far from being confirmed. The correlation between the direct and maternal genetic effects of ADG showed a highly positive correlation. The reports of Dige et al. (25) on Jamunapari goats were positive and consistent for WWT but opposite to that of Menezes et al. (29) and Zhang et al. (4) for Boer goals. Thus, it appears that the selection for WWT or ADG should be based on the direct or maternal genetic effects, whereas the selection for BWT and KR should be based on the direct genetic effect only.

The heritability, maternal heritability, and direct maternal genetic correlation of the same traits between breeds were different. The main reason may be that the breeds were different, and their breeding directions and schemes were also inconsistent, resulting in different micro-effect polygenes affecting the quantitative traits carried inside or outside the nucleus of each breed, thus leading to the genetic diversity of the same quantitative trait among different breeds.

Estimates of different correlations between the investigated traits are shown in Table 6. The absolute values of the direct additive genetic correlations of BWT with other traits were close to zero, which were lower than those of the phenotypic and environmental traits. The low direct additive genetic correlation value of the present study was lower than the BWT-WWT values estimated by Dige et al. (25) for Jamunapari goats, Bangar et al. (30) for Jakhrana goats, and Otuma et al. (41) for Nigeria Sahelian goats. The BWT-ADG and BWT-KR correlation values were estimated to be medium by Dige et al. (25) for Jamunapari goats and by Bangar et al. (30) for Jakhrana goats.

The WWT had a positive and medium genetic correlation with ADG (0.614). Zhou et al. (34) reported a similar value of 0.61 for Hainan Black goats. The direct additive genetic correlation between WWT-KR and ADG-KR were 0.761 and 0.772, which were similar to the estimates by Dige et al. (25) for Jamunapari goats. The high genetic correlation among ADG, WWT, and KR indicated that they were controlled by similar genes and have quite similar genetic factors that influence these traits. This indicated that the selection of one of these traits would have a high genetic advance over the contemporary others. For example, the selection for WWT would have increased ADG and the efficiency of feed utilization in IMWACGs.

However, the maternal genetic correlation was negative between WWT and KR but positive among other traits. The BWT-WWT (0.388), BWT-ADG (0.406), and WWT-ADG (0.577) maternal genetic correlation was medium, while it was high for ADG-KR. The results were significantly lower than those of Dige et al. (25) for Jamunapari goats but consistent with the estimation for Sirohi goats by Gowane et al. (23).

The different genetic correlations between the same traits of different breeds and between different traits of the same breed can be attributed to the following aspects: first, the genes that affect the quantitative traits are mostly micro-effect polygenes that are vulnerable to environmental effects. Different breeds have different breeding environments and selection pressures, resulting in differences in gene expression although they carry the same genes; therefore, the genetic effects of various traits are different in different breeds. Second, according to the principles of genetics, the mechanisms of genetic correlation are pleiotropism and linkage inheritance. Therefore, the number of common genes, the genes for different traits, and the strength of linkage between the genes are the main reasons for the difference in genetic correlation between traits. Finally, the models, data volume, and estimation methods used in statistical analysis are also some important reasons for the differences in parameter estimation between the same traits of different breeds and different traits of the same variety.

The estimates of maternal permanent environmental correlation range from −0.366 (BWT-KR) to 0.957 (WWT-ADG) among early growth traits. The estimates of environmental correlation among WWT, ADG, and KR were positive and high and range from 0.739 for WWT-KR to 0.957 for WWT-ADG, which were probably due to the greater similarity of the environmental and management conditions. The differences in uterine space during pregnancy, nutrients during pregnancy and lactation, maternal behavior, and the mothering ability of the dam may be the main reasons for the variabilities in maternal permanent environmental effects.

The phenotypic correlation estimates among WWT, ADG, and KR changed from −0.294 for BWT–KR to 0.927 for WWT–ADG, which were in agreement with the estimates of environmental correlation for the same traits. The phenotypic correlation estimates between the traits indicated the presence of a desirable association among pre-weaning traits.

## 5. Conclusion

These results indicated that environmental factors have a significant impact on pre-weaning growth traits and hence should be considered while accurately modeling genetic evaluation. The low-to-moderate estimates of direct heritability for pre-weaning growth traits indicated the presence of genetic variabilities within the IMWACGs, and the genetic progress of these traits can be achieved by selection.

The maternal genetic effects and the maternal permanent environmental effects seemed to have a significant effect on early growth traits. Thus, in addition to the direct genetic effect, the maternal genetic effects and maternal permanent environmental effects were found to be important for the genetic evaluation of early growth traits in this breed. The moderate-to-high estimates of genetic correlation among WWT, ADG, and KR indicated that the selection of one of these traits would probably bring out a positive response for the selection from the correlated one. Therefore, selection for early growth traits should only consider one of them when designing a breeding program for this variety.

## Data availability statement

The original contributions presented in the study are included in the article/supplementary material, further inquiries can be directed to the corresponding author.

## Ethics statement

All animal experiments were performed in accordance with the Guidelines for Experimental Animals of the Ministry of Science and Technology (Beijing, China) and were approved by the Scientific Research and Academic Ethics Committee of Inner Mongolia Agricultural University and the Biomedical Research Ethics of Inner Mongolia Agricultural University (Approval No. [2020] 056). Written informed consent was obtained from the owners for the participation of their animals in this study.

## Author contributions

RW, YLiu, and JL conceived the idea and designed the study. RW, ZW, YS, YQ, and YLi analyzed the data. RW and YLiu wrote the draft. YZhan, RS, and YZhao finalized the manuscript. All authors read and approved the final manuscript.

## References

[1] Jiang HZ Guo D Chen Y Zhang SW. Industry status of chinese cashmere goat and analysis of its prospects. Animal Husb Feed Sci. (2009) 30:100–3. 10.3969/j.issn.1672-5190.2009.10.049

[2] Wang Z Wang R Li J Zhang W Su R Liu Z. Modeling genetic covariance structure across ages of fleece traits in an Inner Mongolia cashmere goat population using repeatability and multivariate analysis. Livestock Sci. (2014) 161:1–5. 10.1016/j.livsci.2013.11.028

[3] Guo R. The Effect of Yard-Feeding and Grazing Mode on Meat Quality and Related Indexes of Fat and Protein Metabolism of Arbas Cashmere Goat. Master thesis. Inner Mongolia Agricultural University, Hohhot. (2021).

[4] Zhang CY Zhang Y Xu DQ Li X Su J Yang LG. Genetic and phenotypic parameter estimates for growth traits in Boer goat. Livest Sci. (2009) 124:66–71. 10.1016/j.livsci.2008.12.010

[5] Bai JY Zhang Q Li JQ Dao EJ Jia XP. Estimates of genetic parameters and genetic trends for production traits of inner mongolian white cashmere goat. Asian Austral J Anim Sci. (2006) 19:13. 10.5713/ajas.2006.13

[6] Dai SC Wang CX Wang ZY Wang ZX Zhang YJ Na Q. Inbreeding and its effects on fleece traits of Inner Mongolia cashmere goats. Small Ruminant Research. (2015) 128:50–3. 10.1016/j.smallrumres.2015.04.007

[7] Zhang YJ Wang ZY Hong L Wang ZX Su R Zhang WG. Estimates of genetic parameters and genetic changes for fleece traits in Inner Mongolia cashmere goats. Small Ruminant Research. (2014) 117:41–6. 10.1016/j.smallrumres.2013.10.011

[8] Wang Z Wang R Zhang W Wang Z Wang P Liu H. Estimation of genetic parameters for fleece traits in yearling Inner Mongolia Cashmere goats. Small Ruminant Res. (2013) 109:15–21. 10.1016/j.smallrumres.2012.07.016

[9] Wang ZY Wang ZX Liu Y Wang RJ Zhang YJ Su R. Genetic evaluation of fiber length and fiber diameter from Inner Mongolia White Cashmere goats at different ages. Small Ruminant Res. (2015) 123:22–6. 10.1016/j.smallrumres.2014.11.015

[10] Zhou Allain D Li JQ Zhang WG Yu XC. Effects of non-genetic factors on production traits of Inner Mongolia cashmere goats in China. Small Ruminant Res. (2003) 47:85–9. 10.1016/S0921-4488(02)00246-8

[11] Baneh H Najafi M Rahimi G. Genetic parameter estimates for early growth traits in Naeini goat. Animal Prod Sci. (2012) 52:1046. 10.1071/AN12045

[12] Al-Shorepy SA Alhadrami GA Abdulwahab K. Genetic and phenotypic parameters for early growth traits in Emirati goat. Small Ruminant Res. (2002) 45:217–23. 10.1016/S0921-4488(02)00110-4

[13] Willham RL. The role of maternal effects in animal breeding: III. Biometrical aspects of maternal effects in animals. J Animal Sci. (1972) 35:1288–93. 10.2527/jas1972.3561288x 4567216

[14] Rashidi A Mokhtar MS Safi Jahanshahi A Mohammad Abadi MR. Genetic parameter estimates of pre-weaning growth traits in Kermani sheep. Small Ruminant Res. (2008) 74:165–71. 10.1016/j.smallrumres.2007.06.004

[15] Rashidi A Sheikhahmadi M Rostamzadeh J Shrestha JNB. Genetic and phenotypic parameter estimates of body weight at different ages and yearling fleece weight in markhoz goats. Asian-Aust J Anim Sci. (2008) 21:1395–403. 10.5713/ajas.2008.70752

[16] Boujenanea Hazzab AEI. Genetic parameters for direct and maternal effects on body weights of Draa goats. Small Ruminant Res. (2008) 80:16–21. 10.1016/j.smallrumres.2008.07.026

[17] Mokhtari MS Moghbeli Damaneh M Abdollahi Arpanahi R. The application of recursive multivariate model for genetic evaluation of early growth traits in Raeini Chasmere goat: a comparison with standard multivariate model. Small Ruminant Res. (2018) 4488:30532–7. 10.1016/j.smallrumres.2018.06.008

[18] Gholizadeh M Rahimi Mianji G Hashemi M Hafezian H. Genetic parameter estimates for birth and weaning weights in Raeini goats. Czech J Animal Sci. (2010) 55:30–6. 10.17221/1703-CJAS

[19] Barazandeh A Moghbeli SM Vatankhah M Mohammadabadi M. Estimating non-genetic and genetic parameters of pre-weaning growth traits in Raini Cashmere goat. Trop Anim Health Prod. (2012) 44:811–7. 10.1007/s11250-011-9971-5 21901301

[20] SAS Institute. SAS User's Guide Version 9, 1. Statistics. Cary, NC: SAS Institute Inc.

[21] Meyer K. WOMBAT—A tool for mixed model analyses in quantitative genetics by restricted maximum likelihood (REML). J Zhejiang Univ Sci B. (2007) 8:815–21. 10.1631/jzus.2007.B0815 17973343 PMC2064953

[22] Maghsoudi A VaezTorshizi R SafiJahanshahib A. Estimates of (co) variance components for productive and composite reproductive traits in Iranian Cashmere goats. Livest. Sci. (2009) 126:162–7. 10.1016/j.livsci.2009.06.016

[23] Gowane GR Chopra A Prakash V Arora AL. Estimates of (co)variance components and genetic parameters for growth traits in Sirohi goat. Trop Anim Health Pro. (2011) 43:189–98. 10.1007/s11250-010-9673-4 20711812

[24] Ehsaninia J. Estimates of (co)variance components and genetic parameters for pre-weaning body weight traits and Kleiber ratio in Sangsari sheep breed. Ital J Anim Sci. (2021) 20:918–27. 10.1080/1828051X.2021.1908860

[25] Dige MS Rout PK Singh MK Dass G Kaushik R Gowane GR. Estimation of co (variance) components and genetic parameters for growth and feed efficiency traits in Jamunapari goat. Small Ruminant Res. (2021) 196:106317. 10.1016/j.smallrumres.2021.106317

[26] Yalcin BC Horst P Gerstmayr S Oztan T. Research on the breeding of Angora goats in Turkey. Anim Res Develop. (1989) 30:25–35.

[27] Gerstmayr S Horst P. Estimate of performance traits in Turkish Angora goats. Small Rumin Res. (1995) 16:141–57. 10.1016/0921-4488(95)00631-T

[28] Magotra A Bangar YC Chauhan A Malik BS Malik ZS. Influence of maternal and additive genetic effects on offspring growth traits in Beetal goat. Reprod Domest Animals. (2021) 56:983–91. 10.1111/rda.13940 33884683

[29] Menezes LM Sousa WH Cavalcanti-Filho EP Gama LT. Genetic parameters for reproduction and growth traits in Boer goats in Brazil. Small Ruminant Res. (2016) 136:247–56. 10.1016/j.smallrumres.2016.02.003

[30] Bangar YC Magotra A Yadav AS. Variance components and genetic parameter estimates for pre-weaning and post-weaning growth traits in Jakhrana goat. Small Ruminant Res. (2020) 193:106278. 10.1016/j.smallrumres.2020.106278

[31] Rout PK Matika O Kaushik R Dige MS Dass G Singh MK. Genetic analysis of growth parameters and survival potential of Jamunapari goats in semiarid tropics. Small Ruminant Res. (2022) 165:124–30. 10.1016/j.smallrumres.2018.04.002 30078954 PMC6054051

[32] Sarma L Nahardeka N Goswami RN Aziz A Zaman G Das A Akhtar F. Non-genetic factors affecting pre-weaning growth and morphometric traits in Assam Hill goat. Veterinary World. (2019) 12:1327–31. 10.14202/vetworld.2019.1327-1331 31641315 PMC6755395

[33] Kuthu ZH Javed K Babar ME Sattar A Abdullah M. Estimation of genetic parameters for pre-weaning growth traits in Teddy goats. J Animal Plant Sci. (2017) 27:1408–14.

[34] Zhou HL Gu LH Sun YY Xu TS Rong G. Genetic and phenotypic parameter estimates for growth traits of Hainan Black goat in southern China. Animal Prod Sci. (2015) 55:447–53. 10.1071/AN12228

[35] Portolano B Todaro M Finocchiaro R van Kaam JHBCM. Estimation of the genetic and phenotypic variance of several growth traits of the Sicilian Girgentana goat. Small Ruminant Res. (2002) 45:247–53. 10.1016/S0921-4488(02)00161-X

[36] Tesema Z Alemayehu K Getachew T Kebede D Deribe B Taye M. Estimation of genetic parameters for growth traits and Kleiber ratios in Boer x Central Highland goat. Trop Anim Health Prod. (2020) 52:3195–205. 10.1007/s11250-020-02345-z 32748084

[37] Gupta JP Pandey DP Shah RR. Genetic studies on growth traits of Mehsana goat of Gujarat, India. Indian J Anim Res. (2016) 50:164–7.

[38] Mohammadi H Shahrebabak MM Shahrebabak HM. Genetic parameter estimates for growth traits and prolificacy in Raeini Cashmere goats. Trop Anim Health Prod. (2012) 44:1213–20. 10.1007/s11250-011-0059-z 22213036

[39] Buxadera MA Alexandre G Mandonnet N Navès M Aumont G. Direct genetic and maternal effects affecting litter size, birth weight and pre-weaning losses in Creole goats of Guadeloupe. Animal Science. (2003) 77:363–9. 10.1017/S135772980005431X

[40] Tosh JJ Kemp RA. Estimation of variance components for lamb weights in three sheep populations. J Anim Sci. (1994) 72:1184–90. 10.2527/1994.7251184x 8056662

[41] Otuma MO Osakwe I. Estimation of genetic parameters of growth traits in Nigeria Sahelian goats. J Animal Vet Adv. (2008) 7:535–38. 10.2460/javma.232.9.1362 18447783

